# The Global Burden of Latent Tuberculosis Infection: A Re-estimation Using Mathematical Modelling

**DOI:** 10.1371/journal.pmed.1002152

**Published:** 2016-10-25

**Authors:** Rein M. G. J. Houben, Peter J. Dodd

**Affiliations:** 1 TB Modelling Group, TB Centre, London School of Hygiene and Tropical Medicine, London, United Kingdom; 2 Department of Infectious Disease Epidemiology, London School of Hygiene and Tropical Medicine, London, United Kingdom; 3 School of Health and Related Research, University of Sheffield, Sheffield, United Kingdom; University of California San Francisco, UNITED STATES

## Abstract

**Background:**

The existing estimate of the global burden of latent TB infection (LTBI) as “one-third” of the world population is nearly 20 y old. Given the importance of controlling LTBI as part of the End TB Strategy for eliminating TB by 2050, changes in demography and scientific understanding, and progress in TB control, it is important to re-assess the global burden of LTBI.

**Methods and Findings:**

We constructed trends in annual risk in infection (ARI) for countries between 1934 and 2014 using a combination of direct estimates of ARI from LTBI surveys (131 surveys from 1950 to 2011) and indirect estimates of ARI calculated from World Health Organisation (WHO) estimates of smear positive TB prevalence from 1990 to 2014. Gaussian process regression was used to generate ARIs for country-years without data and to represent uncertainty. Estimated ARI time-series were applied to the demography in each country to calculate the number and proportions of individuals infected, recently infected (infected within 2 y), and recently infected with isoniazid (INH)-resistant strains. Resulting estimates were aggregated by WHO region. We estimated the contribution of existing infections to TB incidence in 2035 and 2050.

In 2014, the global burden of LTBI was 23.0% (95% uncertainty interval [UI]: 20.4%–26.4%), amounting to approximately 1.7 billion people. WHO South-East Asia, Western-Pacific, and Africa regions had the highest prevalence and accounted for around 80% of those with LTBI. Prevalence of recent infection was 0.8% (95% UI: 0.7%–0.9%) of the global population, amounting to 55.5 (95% UI: 48.2–63.8) million individuals currently at high risk of TB disease, of which 10.9% (95% UI:10.2%–11.8%) was isoniazid-resistant. Current LTBI alone, assuming no additional infections from 2015 onwards, would be expected to generate TB incidences in the region of 16.5 per 100,000 per year in 2035 and 8.3 per 100,000 per year in 2050.

Limitations included the quantity and methodological heterogeneity of direct ARI data, and limited evidence to inform on potential clearance of LTBI.

**Conclusions:**

We estimate that approximately 1.7 billion individuals were latently infected with *Mycobacterium tuberculosis* (*M*.*tb*) globally in 2014, just under a quarter of the global population. Investment in new tools to improve diagnosis and treatment of those with LTBI at risk of progressing to disease is urgently needed to address this latent reservoir if the 2050 target of eliminating TB is to be reached.

## Abstract 2

In this mathematical modelling study, Rein Houben and colleagues provide updated estimates of latent tuberculosis infection worldwide and its implications of this reservoir with respect to goals for eliminating tuberculosis.

## Author Summary

**Why Was This Study Done?:**

- Addressing the latent TB infection reservoir is critical to achieving TB elimination.
- The current estimate that “one-third” of the global population is infected with tuberculosis is widely cited but has not been formally estimated for nearly 20 y.
- Changes in demography, the size and distribution of TB burden, as well as new scientific insights and the availability of new data mean a re-estimation is needed.

**What Did the Researchers Do and Find?:**

- We generated an annual risk of infection between 1934 and 2014 and applied this to a country-level demographic model, quantifying uncertainty wherever possible.
- We estimated that approximately 1.7 billion individuals were infected with LTBI in 2014; just under a quarter of the global population.
- If left unaddressed, the current LTBI burden alone will likely prevent achieving the global TB targets for TB elimination.

**What Do These Findings Mean?:**

- For long-term TB control to be successful, an aggressive approach to LTBI is needed.
- Research and development should focus on developing better tools to identify individuals who will benefit from LTBI treatment.
- Estimates would be strengthened by additional empirical data from new population-based studies of LTBI prevalence.

## Introduction

Infection with *Mycobacterium tuberculosis* (*M*.*tb*) is the precursor to TB disease, which is responsible for 1.5 million deaths each year—more than any other infectious disease [1]. Once infected, the individual is at highest risk of developing TB disease within the first two years, but can remain at risk for their lifetime [2]. The population carrying a latent TB infection (LTBI) is commonly quoted as “one-third” of the global population, a reservoir of approximately 2.3 billion individuals [3–6].

As the global community looks to meet ambitious targets for reduction (90% reduction in TB incidence by 2035) and even elimination of TB (less than 1 incident case per 1,000,000 per year) by 2050 [7], our ability to address the LTBI reservoir will be critical in our chance to succeed.

Despite its clear importance to global TB control efforts, the most recent attempt to estimate the global burden of LTBI was in 1998 [3]. Since then, the size and distribution of the global population [8] and TB burden [1] has changed dramatically, as has our understanding of prevalent disease as a driver of infection [9,10]. Global population growth from around 6 billion in 1998 to over 7 billion in 2014 has been mainly driven by areas with the highest TB burden, such as Southeast Asia and sub-Saharan Africa [1,8]. The previous estimation method relied on a fixed relationship between TB burden to the annual risk of acquiring LTBI, the so-called “Styblo rule” [3]. Since then, two groups have shown this long-held rule of thumb substantially overestimates infection risk in modern populations [9,10].

Given these changes and the drive towards eliminating TB [7,11], an updated estimate of the global burden of LTBI that incorporates the available data and applies current scientific insights is urgently needed [4,12].

An updated estimate of the size and distribution of the LTBI reservoir should also address questions about the likely contribution of LTBI to TB disease over the coming decades. Specifically, how many active TB cases would arise from the currently infected individuals alone if all transmission was halted now? Updated LTBI burden estimates also indicate the population in need of interventions and new tools, thus catalyzing new research and potential investment from commercial partners in, for example, vaccines, and tools for the diagnosis and treatment of LTBI [4].

Critical questions include the number of those with LTBI at highest risk of developing disease, i.e., those infected within the past 2 y [12]. This population is a focus of proposed “test and treat” strategies in TB, which would use an RNA expression profile test to identify individuals most likely to develop TB [13], improving on the low predictive value of existing tests for LTBI [14]. As resistance to TB drugs is rising, an estimate of the proportion of LTBI that involves isoniazid (INH)-resistant strains is important, since INH remains the cornerstone of most treatment regimens for LTBI [4]. Finally, as TB becomes rarer, the epidemiology of LTBI will have a renewed and increasing importance for monitoring the progress of control efforts.

In this paper, we estimate the global burden of LTBI and its distribution by country, geographical region, and age group. We also estimate the number of recent infections and the number of recent infections with INH-resistant strains. Finally, we predict the TB incidence in 2035 and 2050 solely due to the existing LTBI reservoir.

## Methods

To estimate the burden of LTBI, we reconstructed country trends in annual risk of *M*.*tb* infection (ARI) and combined these historical projections with demographic data to estimate burden of recent and all-time infection by age. ARI trends were modelled for 168 countries (comprising >99.9% of the world population) using a flexible non-parametric regression framework accounting for measurement uncertainty and applied to two sources of data.

The first source of data was direct estimates of ARI from tuberculin skin test (TST) surveys. We abstracted data on estimated ARI, survey sample size, and mean age for 100 country-years in 24 countries from Cauthen et al. [15] and undertook a systematic review of nationally representative ARI estimates in the years 1990–2014 (see Methods and Figure A in S1 Text), yielding further data on 31 country-years in 19 countries, to give a total of 37 unique countries with TST survey data. Historically, ARIs have been estimated from TST surveys without presentation of uncertainty. In the Methods section of S1 Text, we show how sample size and mean age can be used to approximately and conservatively quantify measurement precision. We used this method for studies not stating ARI estimate precision.

The second source of data was indirect: combining WHO estimates of TB prevalence (5,373 country-years for 218 countries) with an uncertain representation of the revised Styblo ratio that accounts for uncertainty and relates the prevalence of smear positive disease to ARI [1,9,10]. A previously published study of childhood tuberculosis [16] characterized this ratio in the modern era by fitting a log-normal distribution to data from reviews of studies in which both ARI and prevalence estimates were available [9,10]. To estimate the proportion of prevalent TB that is smear positive for each country, we averaged estimates of smear positivity for 0–4, 5–14, and ≥15 y age groups from a recent systematic review [17] against the proportion of cases in these age-groups calculated using the model of Dodd et al. [16]. To calculate the impact of HIV on the proportion of TB in a country that is smear positive, we first calculated the fraction of prevalent TB in people living with HIV (PLHIV) by adjusting the WHO estimates of HIV prevalence in incident TB for each year using estimates of the duration of prevalence in PLHIV and HIV-uninfected individuals [1]. The mean smear positivity of HIV-TB was then reduced by a fraction reported in Corbett et al. [18]. The uncertainty of each ingredient in these ARI calculations was propagated using the delta method. More details are described in the Methods section of S1 Text.

Gaussian process regression with a linear trend was applied to the data on ARI (on a log scale), using the measurement precision calculated for each data point. This implicitly combines the data feeding into WHO prevalence estimates (and Styblo ratio) and the TST data feeding into ARI estimates, with the assumption of a normal approximation to the likelihood. A sensitivity analysis re-analysed these data with a constant rather than linear trend assumption. To allow a comparison with the 1998 estimate, we assumed a constant ARI before 1934.

For each country, 200 simulated ARI trajectories from 1934 to 2014 were used to compute the cumulative hazard of infection for individuals by age. The cumulative hazard was converted into a probability of infection and combined with UN Population Division estimates of country demography in 2014 to give our estimates of all-time infection. We computed the probability of infection for the first time within 2 y, using the cumulative hazard up to 2012 to calculate the fraction at each age who had escaped infection until then, and the cumulative hazard from 2012 to 2014 to calculate the fraction of these who were then infected. To calculate the fraction of those at a given age infected or re-infected during the last 2 y, we introduced a beta distribution characterizing an uncertain partial protection against re-infection of 79% (70%–86%) from Andrews et al. [19]. See the Methods section of S1 Text for details. As estimates of protection have varied [20,21], we conducted a sensitivity analysis using a protection of 50% with the same variance. Estimates of infection prevalence were summarized by region, age, and medians mapped by country.

To calculate the number of infections within the last 2 y with resistance to isoniazid, we combined our estimates of infection within 2 y in each country with a recent analysis of the proportion of new infections in each country that are isoniazid-resistance using data from the Global Project on Anti-tuberculosis Drug Resistance Surveillance at WHO [22]. This proportion was treated as uncertain and sampled from the output of this analysis. A conceptual overview diagram for the methods is presented in Figure R in S1 Text.

Finally, we estimated the regional prevalence of latent infection in 2035 and 2050 under the assumption of no *M*.*tb* transmission after 2014, using UN Population Division demographic projections, and calculated the likely implications of existing *M*.*tb* infections for future TB incidence, assuming a 0.15% per year remote activation rate [21,23,24].

Results are reported as medians together with 95% uncertainty intervals (95% UI), calculated as the 2.5% to 97.5% percentile range. No specific funding was received for this work.

## Results

### ARI Estimates

**Fig 1:**
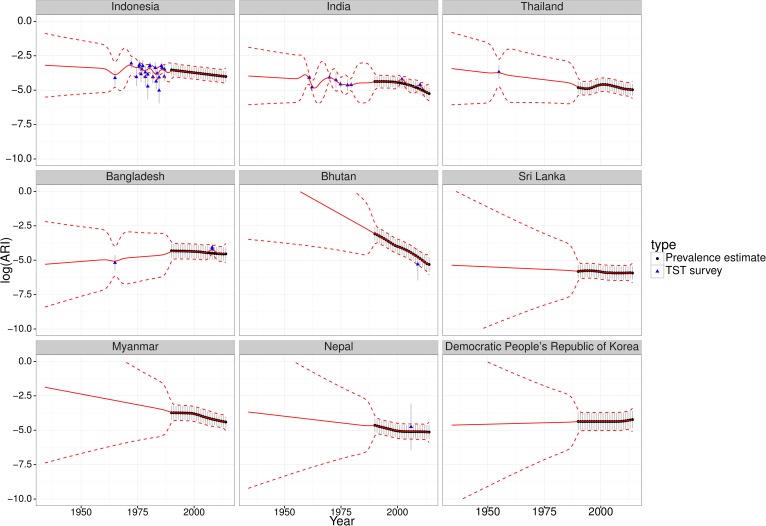
Fitted trends for annual risk of infection—Southeast Asia. Fitted trends of annual risk of tuberculosis infection (ARI) for countries in the WHO Southeast Asia region (log scale). Points represent ARI data (black circles from WHO tuberculosis prevalence estimates, blue triangles from tuberculin skin test [TST] surveys); error bars represent measurement precision to +/- one standard deviation. Red lines show the mean (solid) and +/- one standard deviation (dashed lines) of Gaussian process regression with a linear trend. Similar plots for the other WHO regions are provided in S1 Text (Figures C-H).

The ARI estimates from TST surveys were comparable with the ARI estimates from WHO TB prevalence estimates via the updated Styblo rule and typically did not exhibit discontinuities (Figures C–H in S1 Text). Fig 1 shows the results for the WHO Southeast Asia region. Fig 1 also illustrates uncertainty increasing at earlier times, away from data.

### LTBI Prevalence on Global, Regional, and Country Level

**Fig 2:**
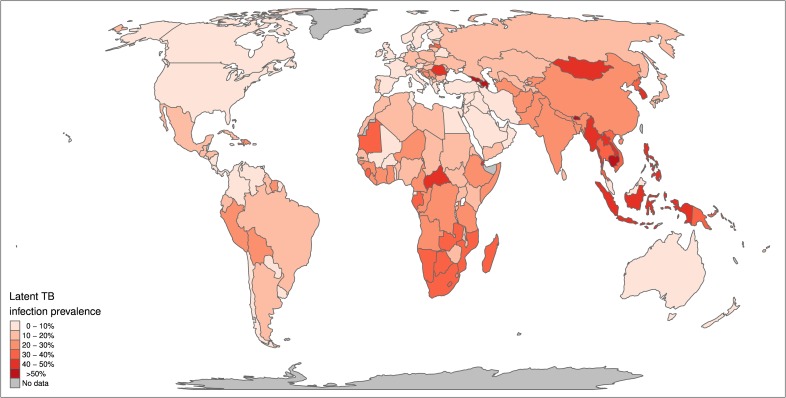
Global map of prevalence of latent TB infection. Median estimated population prevalence of latent *Mycobacterium tuberculosis* infection by country, 2014.

We estimate a global prevalence of latent *M*.*tb* infection in 2014 of 23.0% (95% UI: 20.4%–26.4%) (Table 1). This amounts to an estimate for the worldwide prevalence of *M*.*tb* infection of 1.7 billion (95% UI: 1.5 billion–1.9 billion) in 2014 (Table 2). Fig 2 highlights the substantial regional and sub-regional variation in LTBI prevalence. The WHO Southeast Asia, Western Pacific, and Africa regions were all estimated to have LTBI prevalence in the general population of above around 20% (see first column of Table 1), whereas the WHO Eastern-Mediterranean, Europe, and Americas regions all had general population LTBI prevalence of below 17%. The large populations and high proportion infected imply that around 80% of the number of people with latent infection are in the WHO Southeast Asia, Western Pacific, and Africa regions, compared to 65% of the total population. On the country level, China and India had the highest LTBI burden, approximately 350 million infections, followed by Indonesia at around 120 million infections and fewer than 60 million infections in all other countries. The USA had the 20th highest burden, at an estimated 13 million (Figure J in S1 Text).

**Table 1:** Proportion of population with latent TB infection.

WHO region	All LTBI		Recent infection prevalence (within 2 y)	
	Prevalence (%)	Proportion of infections in children <15 y (%)	(%)	Proportion with INH-R infection (%)
AFR	22.4 [20.6–24.6]	13.3 [11.8–14.6]	1.5 [1.3–1.7]	7.4 [6.4–8.7]
AMR	11.0 [7.0–20.0]	2.3 [1.3–3.7]	0.2 [0.1–0.2]	7.0 [6.0–8.8]
SEA	30.8 [28.3–34.8]	7.4 [6.3–8.2]	1.2 [0.9–1.6]	9.5 [8.8–10.3]
EMR	16.3 [13.4–20.5]	7.9 [6.0–9.4]	0.7 [0.5–1.0]	13.1 [10.0–15.5]
WPR	27.9 [19.3–40.1]	2.4 [1.7–3.5]	0.5 [0.4–0.7]	14.7 [13.9–15.6]
EUR	13.7 [9.8–19.8]	2.0 [1.3–2.7]	0.3 [0.2–0.3]	29.5 [23.8–45.1]
**GLOBAL**	23.0 [20.4–26.4]	5.9 [5.1–6.7]	0.8 [0.7–0.9]	10.9 [10.2–11.8]

Proportion of population by WHO region infected with *Mycobacterium tuberculosis*, 2014 (including proportion of LTBI burden that is in children, proportion recently infected, and proportion of recent infections with isoniazid-resistant (INH-R) *Mycobacterium tuberculosis*). Brackets indicate 95% uncertainty interval. AFR = African Region; AMR = Region of the Americas; EMR = Eastern Mediterranean Region; EUR = European Region; SEA = Southeast Asia Region; WPR = Western Pacific Region

**Table 2:** Number (thousands) of individuals with latent TB infection

WHO region	All LTBI		Recent infection prevalence (within 2 y)	
	Number (K)	Number (K) of infections in children <15 y	Number (K)	Number (K) with INH-R infection (%)
AFR	216,000 [198,000–237,000]	28,700 [26,700–30,800]	14,300 [12,200–16,800]	1,060 [844–1,310]
AMR	108,000 [68,900–196,000]	2,470 [2,240–2,710]	1,760 [1,440–2,240]	126 [99–162]
SEA	587,000 [540,000–662,000]	43,300 [38,700–48,300]	23,000 [17,100–30,900]	2,210 [1,650–2,950]
EMR	104,000 [85,200–130,000]	8,060 [7,090–9,240]	4,520 [3,160–6,280]	581 [386–847]
WPR	514,000 [356,000–739,000]	12,400 [10,900–13,800]	9,130 [6,800–12,900]	1,340 [1,000–1,940]
EUR	124,000 [89,100–180,000]	2,430 [2,220–2,690]	2,300 [1,860–3,120]	686 [461–1,200]
**GLOBAL**	**1,660,000 [1,480,000**–**1,910,000]**	**97,100 [91,700**–**103,000]**	**55,500 [48,200**–**63,800]**	**6,060 [5,140**–**7,040]**

Number of individuals by WHO region infected with *Mycobacterium tuberculosis*, 2014 (including proportion of LTBI burden that is in children, proportion recently infected and proportion of recent infections with isoniazid resistant (INH-R) *Mycobacterium tuberculosis*). Numbers in thousands (K). Brackets indicate 95% uncertainty interval. AFR = African Region; AMR = Region of the Americas; EMR = Eastern Mediterranean Region; EUR = European Region; SEA = Southeast Asia Region; WPR = Western Pacific Region

### Age Trends

**Fig 3:**
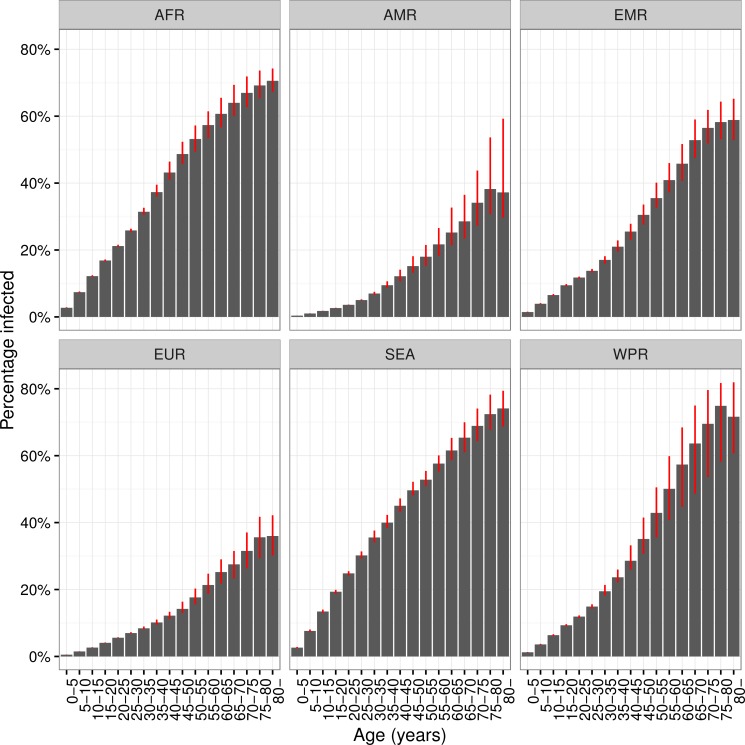
Prevalence of latent TB infection by age and WHO region. Prevalence of latent *Mycobacterium tuberculosis* infection by age and World Health Organisation region, 2014. Red error bars indicate inter-quartile range. AFR = African Region; AMR = Region of the Americas; EMR = Eastern Mediterranean Region; EUR = European Region; SEA = Southeast Asia Region; WPR = Western Pacific Region.

The proportions of each age group infected by region are shown in Fig 3, and column 2 in Table 2 shows the percentage of all LTBI in children under 15 y old. While around 6% of *M*.*tb* infections are in children globally, 13% of infections in Africa are in children, compared to 2% in the Americas. In all regions, the proportion infected rises with age and, with the exception of the WHO Europe and Americas regions, exceeds 50% in the oldest age groups. The substantial increases in TB burden in Africa and Southeast Asia are reflected in the shape of Fig 3, with more rapid increases in the younger age groups compared to other regions. Uncertainty in these proportions is largest for the Western Pacific region, particularly in older age groups, due to larger uncertainty in historical ARIs there.

### Recent Infection and Isoniazid Resistance

Around 1% of the global population, approximately 56 million individuals, was infected within the last 2 y and would therefore be at appreciable risk of progressing to active TB (see Tables 1 and 2). Of these recent infections, the vast majority are infections for the first time; however, this, too, varies by age (Figure J in S1 Text). We estimate that around 11% of these recent infections involved an isoniazid-resistant strain of *M*.*tb*, amounting to around 6 million individuals at elevated risk of TB in whom isoniazid-preventive therapy would be ineffective (see Tables 1 and 2). There is strong regional variation, with around 30% of the recent infections in the European region involving an isoniazid-resistant strain.

### Current Burden of LTBI and End TB Strategy Targets

If we assume no ongoing transmission from 2015 onwards, our projections of current LTBI burden imply that in 2035, 961 (95% UI: 870–1,113) million individuals would still be infected (around 11% of the population). By 2050, these numbers would be 599 (95% UI: 557–668) million (around 6%). Assuming a remote LTBI activation rate of 0.15% per year, this implies a TB disease incidence from these latent pools of 16.5 (95% UI: 14.9–19.2) per 100,000 per year in 2035, which is above the 10 per 100,000 per year target in the End TB Strategy. In 2050, the rate would be 8.3 (95% UI: 8.6–10.6) per 100,000 per year; nearly two orders of magnitude higher than the 2050 elimination target of 1 per million per year.

### Sensitivity Analyses

Sensitivity analysis assuming constant trends in the Gaussian process regression (Figures L–Q in S1 Text) led to smaller estimates of LTBI burden due to lower extrapolated ARIs at earlier times. In general, estimates in prevalence were around 20% lower with this assumption, giving global estimates of 18.5% (95% UI: 17.0%–20.7%) LTBI prevalence or 1.3 (95%UI: 1.2–1.5) billion infected individuals. Considering a lower protection against reinfection of 50% made little difference to overall numbers but resulted in more recent infections at older age groups (see Tables A–D and Figure Q in S1 Text). Applying our method to estimate the population in 1997 yielded an LTBI prevalence of 26.9% (95% UI: 22.4%–32.7%).

## Discussion

The global burden of LTBI is just under a quarter of the population—around 1.7 billion individuals—with substantial geographical and age variation. We estimate 56 million people are at high risk of developing TB disease because of a recent (re-)infection, 11% of whom are carrying an isoniazid-resistant strain. With reasonable assumptions for reactivation risks, incident TB disease arising from the 2014 LTBI reservoir alone would prohibit reaching the 2035 and 2050 End TB Strategy goals.

Using our method to calculate LTBI prevalence in 1997 yielded 27%, suggesting the difference between our estimate for 2014 and the 1997 estimate [3] is mainly due to changes in methodology, including the revised Styblo rule [9,10]. Our results also matched a recent survey-based estimate of 13 million latent infections for the United States [25].

One limitation of this study was that we were not able to consider the effects due to different tuberculin strains and cut-off choices for TST tests or the potential impact of BCG vaccination on these test outcomes. Recent work has suggested that LTBI may be a dynamic and heterogeneous state [12] and highlighted the difference between *M*.*tb* infection and positive tests for infection. Our work does not make this distinction but is in line with literature relating TB prevalence to infection risk and risks of progression to disease. In addition, we assumed lifelong LTBI in common with previous estimates and consistent with observation [2]. While it may be biologically plausible that some individuals clear their LTBI in absence of treatment, there is no published evidence to support a separate model scenario.

Our approach to quantifying the typical infectiousness of prevalent TB cases by country entailed some necessary simplifications. Notably, we assumed the same TB case-detection rate applied independent of HIV status and anti-retroviral treatment status. However, these assumptions had relatively little influence on ARI estimates and their uncertainty (see S1 Text, page 8), with the dominant contributions to uncertainty coming from the TB prevalence estimates themselves and uncertainty in the Styblo ratio. We also assumed that the same ARI applied to all individuals in a country and neglected migration between countries.

A major strength of this study is its treatment of uncertainty. We were able to characterize and include measurement precision for data on ARI derived from TST surveys as well as indirectly from prevalence estimates. While uncertainty in ARI estimates grew substantially for the earliest years, the number of people alive today who were alive then was small, limiting the impact of this uncertainty on our overall estimate. This also limited the impact of our assumption of linear trends extrapolating backward from data; our very conservative assumption of flat trends resulted in less than a five-percentage-point difference in our overall prevalence estimate.

Our estimate of TB incidence in 2035 and 2050 from currently existing LTBI uses a single reactivation rate. This parameter has been estimated at widely varying levels, [23,24,26], and we have not attempted to include potential geographic heterogeneity or anticipate trends due to changes in population health, for example, through achievements of the Sustainable Development Goals (SDG) [27] or due to cohort aging. However, our estimates of TB disease incidence in 2035 and 2050 are proportional to this parameter, and even large reductions do not alter the conclusion that incidence will exceed the 2050 elimination target.

It is a limitation that there were not more recent direct national estimates of ARI, which would have strengthened our estimates. More empirical data on the epidemiology of LTBI, including from the use of modern tests that are less prone to biases of interpretation and cross-reaction [28], should improve our understanding of these features and generate more data upon which to base estimates. As active TB becomes rarer, surveys of infection will become an increasingly appealing option for monitoring trends, but this relies on understanding the relationship between infection and other burden metrics.

Policies to address the LTBI reservoir have to balance the potential of harm versus the benefit for the individual [29]. In the current landscape of diagnostic tools, WHO recommends LTBI testing and treatment only in high-risk groups, such as people living with HIV, and close contacts of TB cases. While these guidelines are sensible, it is clear that a more aggressive approach is needed to reduce the threat to long-term TB control targets stemming from a LTBI reservoir of approximately 1.7 billion individuals. Future work with this model could inform current policies by estimating the burden of LTBI in specific risk groups, such as people living with HIV or diabetes. A test to more precisely identify those at substantial risk of progressing to disease could enable targeted LTBI treatment beyond known risk groups [12]. Emerging tests based on RNA signatures may come to provide a more practicable method of identifying individuals for LTBI treatment [13]. Among biomedical interventions, a vaccine that prevents progression to disease from LTBI could make a major contribution, depending on global availability [30]. Beyond the biomedical perspective, improvements in social and economic conditions globally have been associated with reductions in TB burden in historic and contemporary contexts [31,32], and could also contribute to reducing the TB burden originating from the LTBI reservoir.

Treatment for LTBI still relies heavily on isoniazid, either as monotherapy or as part of a combination regimen [4,11]. We found that just under 11% of all recent *M*.*tb* infections are likely to be isoniazid resistant, with much higher rates in some regions, and this proportion is likely to increase. While less common, rifampicin resistance also has the potential to threaten the usefulness of rifampicin-containing prophylactic regimens. New treatments that bypass the rising resistance to isoniazid and rifampicin are needed to fully operationalise interventions to test and treat LTBI.

## Conclusion

We estimate that approximately 1.7 billion individuals were latently infected with *M*.*tb* globally in 2014, just under a quarter of the global population. Investment in new tools to improve diagnosis and treatment of those with LTBI at risk of progressing to disease is urgently needed to address this latent reservoir if the TB community is to reach the 2050 target of eliminating TB.

## Supporting Information

S1 Data WHO Global TB Programme prevalence estimates for each country (1990–2014). Global TB Programme prevalence estimates used in the model.
(CSV)

S2 Data ARI estimates extracted from Cauthen et al. Estimates for annual risk of infection (ARI) used in the model, providing country, year, ARI, number of individuals in survey, and age.
(CSV)

S3 Data ARI estimates extracted from literature review (1990–current). Estimates for annual risk of infection (ARI) used in the model, providing country, year, ARI, number of individuals in survey, and age.
(CSV)

S1 GATHER Checklist GATHER Checklist. (DOCX)

S1 PRISMA Checklist PRISMA Checklist. (DOC)

S1 Text Supporting information. Supporting information on systematic review, methods, additional results, and sensitivity analyses.
(DOCX)

S1 Results Estimates of population with LTBI in 2014 by country. Individual country estimates for LTBI prevalence in 2014. Note: country-level estimates are more subject to bias and uncertainty and should be used with caution. Brackets show IQR; point estimate is the mean.
(CSV)

## Abbreviations

ARI: annual risk in infection
INH: isoniazid
LTBI: latent TB infection
M.tb: Mycobacterium tuberculosis
PLHIV: people living with HIV
SDG: Sustainable Development Goals
TST: tuberculin skin test
WHO: World Health Organisation

## References

[1] World Health Organisation. Global Tuberculosis Report 2015. Geneva: 2015. http://www.who.int/tb/publications/global_report/en/ (Accessed 23 September 2016)

[2] Lillebaek T, Dirksen A, Baess I, Strunge B, Thomsen VO, Andersen AB. Molecular evidence of endogenous reactivation of Mycobacterium tuberculosis after 33 years of latent infection. J Infect Dis. 2002;185(3):401–4. 10.1086/338342 . 11807725

[3] Dye C, Scheele S, Dolin P, Pathania V, Raviglione MC. Consensus statement. Global burden of tuberculosis: estimated incidence, prevalence, and mortality by country. WHO Global Surveillance and Monitoring Project. JAMA: the journal of the American Medical Association. 1999;282(7):677–86. Epub 1999/10/12. . 10517722 10.1001/jama.282.7.677

[4] Getahun H, Matteelli A, Chaisson RE, Raviglione M. Latent Mycobacterium tuberculosis infection. N Engl J Med. 2015;372(22):2127–35. 10.1056/NEJMra1405427 . 26017823

[5] World Health Organisation. Tuberculosis Fact Sheet 2015 [1st March 2016]. http://www.who.int/mediacentre/factsheets/fs104/en/

[6] Centers for Disease Control (USA). Tuberculosis—Data and Statistics 2015 [1st March 2016]. http://www.cdc.gov/tb/statistics/default.htm.

[7] Uplekar M, Weil D, Lonnroth K, Jaramillo E, Lienhardt C, Dias HM, et al WHO's new End TB Strategy. Lancet. 2015;385:1799–801. 10.1016/S0140-6736(15)60570-010.1016/S0140-6736(15)60570-0 Epub 2015 Mar 24. . 25814376

[8] United Nations—Department of Economic and Social Affairs—Population Division. World Population Prospects: The 2015 Revision, Key Findings and Advance Tables. Working Paper No. ESA/P/WP.241. 2015.

[9] van Leth F, van der Werf MJ, Borgdorff MW. Prevalence of tuberculous infection and incidence of tuberculosis: a re-assessment of the Styblo rule. Bull World Health Organ. 2008;86(1):20–6. Epub 2008/02/01. 10.2471/blt.06.037804 18235886 PMC2647347

[10] Trunz BB, Fine P, Dye C. Effect of BCG vaccination on childhood tuberculous meningitis and miliary tuberculosis worldwide: a meta-analysis and assessment of cost-effectiveness. Lancet. 2006;367(9517):1173–80. 10.1016/S0140-6736(06)68507-3 . 16616560

[11] World Health Organisation. Framework towards TB elimination in low-incidence countries Geneva: World Health Organisation, 2014 http://www.who.int/tb/publications/elimination_framework/en/ (Accessed 23 September 2016)

[12] Esmail H, Barry CE 3rd, Young DB, Wilkinson RJ. The ongoing challenge of latent tuberculosis. Philosophical transactions of the Royal Society of London Series B, Biological sciences. 2014;369(1645):20130437 Epub 2014/05/14. 10.1098/rstb.2013.0437 24821923 PMC4024230

[13] Zak DE, Penn-Nicholson A, Scriba TJ, Thompson E, Suliman S, Amon LM, et al A blood RNA signature for tuberculosis disease risk: a prospective cohort study. The Lancet. 2016 10.1016/S0140-6736(15)01316-1 27017310 PMC5392204

[14] Rangaka MX, Wilkinson KA, Glynn JR, Ling D, Menzies D, Mwansa-Kambafwile J, et al Predictive value of interferon-gamma release assays for incident active tuberculosis: a systematic review and meta-analysis. Lancet Infect Dis. 2012;12(1):45–55. Epub 2011/08/19. 10.1016/S1473-3099(11)70210-9 . 21846592 PMC3568693

[15] Cauthen GM, Pio A, ten Dam HG. Annual risk of tuberculous infection. 1988. Bull World Health Organ. 2002;80(6):503–11; discussion 1–2. Epub 2002/07/20. 12132011 PMC2567543

[16] Dodd PJ, Gardiner E, Coghlan R, Seddon JA. Burden of childhood tuberculosis in 22 high-burden countries: a mathematical modelling study. The Lancet Global health. 2014;2(8):e453–9. 10.1016/S2214-109X(14)70245-1 . 25103518

[17] Kunkel A, Abel Zur Wiesch P, Nathavitharana RR, Marx FM, Jenkins HE, Cohen T. Smear positivity in paediatric and adult tuberculosis: systematic review and meta-analysis. BMC Infect Dis. 2016;16(1):282 10.1186/s12879-016-1617-9 27296716 PMC4906576

[18] Corbett EL, Watt CJ, Walker N, Maher D, Williams BG, Raviglione MC, et al The growing burden of tuberculosis: global trends and interactions with the HIV epidemic. Arch Intern Med. 2003;163(9):1009–21. 10.1001/archinte.163.9.1009 . 12742798

[19] Andrews JR, Noubary F, Walensky RP, Cerda R, Losina E, Horsburgh CR. Risk of progression to active tuberculosis following reinfection with Mycobacterium tuberculosis. Clin Infect Dis. 2012;54(6):784–91. Epub 2012/01/24. 10.1093/cid/cir951 22267721 PMC3284215

[20] Sutherland I, Svandova E, Radhakrishna S. The development of clinical tuberculosis following infection with tubercle bacilli. Tubercle. 1982;63(4):255–68. 10.1016/s0041-3879(82)80013-5 . 6763793

[21] Vynnycky E, Fine PE. The natural history of tuberculosis: the implications of age-dependent risks of disease and the role of reinfection. Epidemiol Infect. 1997;119(2):183–201. 10.1017/s0950268897007917 . 9363017 PMC2808840

[22] Dodd PJ, Sismanidis C, Seddon JA. Global burden of drug-resistant tuberculosis in children: a mathematical modelling study. The Lancet Infectious Diseases. 2016 10.1016/S1473-3099(16)30132-3 PubMed Central PMCID: PMC27342768. 27342768

[23] Ferebee SH, Mount FW, Murray FJ, Livesay VT. A Controlled Trial of Isoniazid Prophylaxis in Mental Institutions. The American review of respiratory disease. 1963;88:161–75. 10.1164/arrd.1963.88.2.161 . 14045220

[24] Sloot R, Schim van der Loeff MF, Kouw PM, Borgdorff MW. Risk of tuberculosis after recent exposure. A 10-year follow-up study of contacts in Amsterdam. Am J Respir Crit Care Med. 2014;190(9):1044–52. 10.1164/rccm.201406-1159OC . 25265362

[25] Mancuso JD, Diffenderfer JM, Ghassemieh BJ, Horne DJ, Kao TC. The Prevalence of Latent Tuberculosis Infection in the United States. Am J Respir Crit Care Med. 2016 Epub 2016/02/13. 10.1164/rccm.201508-1683OC . 26866439 PMC12057332

[26] Horsburgh CR Jr., O'Donnell M, Chamblee S, Moreland JL, Johnson J, Marsh BJ, et al Revisiting rates of reactivation tuberculosis: a population-based approach. Am J Respir Crit Care Med. 2010;182(3):420–5. 10.1164/rccm.200909-1355OC 20395560 PMC2921602

[27] United Nations. Sustainable Development Goals 2015 [17-April-2015]. https://sustainabledevelopment.un.org/topics/sustainabledevelopmentgoals.

[28] Pai M, Denkinger CM, Kik SV, Rangaka MX, Zwerling A, Oxlade O, et al Gamma interferon release assays for detection of Mycobacterium tuberculosis infection. Clinical microbiology reviews. 2014;27(1):3–20. 10.1128/CMR.00034-13 24396134 PMC3910908

[29] World Health Organisation. Guidelines on the Management of Latent Tuberculosis Infection Geneva: World Health Organization Copyright (c) World Health Organization 2015., 2015 http://www.who.int/tb/publications/latent-tuberculosis-infection/en/ (Accessed on 23 September 2016)

[30] Abu-Raddad LJ, Sabatelli L, Achterberg JT, Sugimoto JD, Longini IM Jr., Dye C, et al Epidemiological benefits of more-effective tuberculosis vaccines, drugs, and diagnostics. Proceedings of the National Academy of Sciences of the United States of America. 2009;106(33):13980–5. Epub 2009/08/12. 0901720106 [pii] 10.1073/pnas.0901720106 19666590 PMC2720405

[31] Janssens JP, Rieder HL. An ecological analysis of incidence of tuberculosis and per capita gross domestic product. Eur Respir J. 2008;32(5):1415–6. 10.1183/09031936.00078708 . 18978146

[32] Dye C, Lonnroth K, Jaramillo E, Williams BG, Raviglione M. Trends in tuberculosis incidence and their determinants in 134 countries. Bull World Health Organ. 2009;87(9):683–91. 10.2471/blt.08.058453 19784448 PMC2739916

