# Supplementary material for: The Global Burden of Latent Tuberculosis Infection: A Re-estimation Using Mathematical Modelling
Source: PLoS Med. 2016 Oct 25;13(10):e1002152. doi: 10.1371/journal.pmed.1002152 (PMC5079585; doi:10.1371/journal.pmed.1002152)
Supplement: S1 Text — Supporting information on systematic review, methods, additional results, and sensitivity analyses. (DOCX) [file pmed.1002152.s006.docx]

**The global burden of latent tuberculosis infection**

**(supporting information)**

Rein M. G. J. Houben^1,2^ and Peter J. Dodd^3^

1. TB Modelling Group, TB Centre, London School of Hygiene and Tropical Medicine, London, UK
2. Department of Infectious Disease Epidemiology, London School of Hygiene and Tropical Medicine, London, UK
3. School of Health and Related Research, University of Sheffield, Sheffield, UK

Methods 2

Systematic review 2

Literature search 2

Figure A: PRISMA flow chart for systematic review 3

References from review (ordered by time of publication) 4

Uncertainty in ARI estimates from TST surveys 6

Data merging and special cases 6

Figure B: Ranked country sizes (500,000 population size cut-off as red line) 7

Modelling ARI from TB prevalence estimates 7

The Styblo ratio 7

The influence of HIV on smear-positivity 8

The influence of age mix on smear-positivity 8

Propagation of uncertainty 9

Gaussian Process calculations 9

Modelling LTBI burden given ARI 9

All M.tb infection 9

Infection with M.tb for the first time within 2 years 10

Infection or re-infection with M.tb within 2 years 10

Results 12

Gaussian process regressions for ARI with linear trends 12

Figure C: WHO AFR region 12

Figure D: WHO AMR region 13

Figure E: WHO EMR region 14

Figure F: WHO EUR region 15

Figure G: WHO SEA region 16

Figure H: WHO WPR region 17

Top 20 countries by absolute LTBI burden (figure) and country level results (file) 18

Figure I: Top 20 countries by number of latent *M.tb* infections (color shows percentage prevalence) 18

Recent infection and re-infection by age 19

Figure J: Recent infection and re-infection by age under base case assumption around protection from re-infection. 19

Gaussian process regressions for ARI with flat trends 20

Figure K: WHO AFR region (flat trends) 20

Figure L: WHO AMR region (flat trends) 21

Figure M: WHO EMR region (flat trends) 22

Figure N: WHO EUR region (flat trends) 23

Figure O: WHO SEA region (flat trends) 24

Figure P: WHO WPR region (flat trends) 25

Supporting tables 26

Table A: Proportions with LTBI for flat trend sensitivity analysis 26

Table B: Proportions with LTBI for 50% protection sensitivity analysis 26

Table C: Numbers (thousands) of infections for flat trend sensitivity analysis 27

Table D: Numbers (thousands) of infections for 50% protection sensitivity analysis 27

Recent infection and re-infection by age (sensitivity analysis) 28

Figure Q: Sensitivity analysis 50% protection against re-infection 28

Conceptual overview 29

Figure R: Conceptual overview 29

# Methods

## Systematic review

### Literature search

We searched the literature for papers reporting on nationally representative LTBI survey results and provide an estimate of the ARI for that country. In addition we scanned references from two papers that explored the Styblo rule (Bourdin-Trunz *et al*. and van Leth *et al.* The focus of the search was to identify data to supplement the period where our ARI estimates relied on GTB estimates of prevalence, i.e. 1990 onwards. This review was not registered in a formal database.

Papers were included if the aim of their sampling method was to provide a representative national level estimate, so they could be combined with the existing data from existing reviews.

To be included, papers had to report the minimum required data needed in the model, which included year of survey, average age or range of years in the cohort, the number of participants in the analysis, the number positive or prevalence of infection and the estimated annual risk of infection. If the number tested and positive were missing, an estimate of the ARI with a 95% confidence interval was also acceptable.

We restricted the search to papers available through online databases. If the required data was not available from the abstract, electronic full text was searched for. If this was not available, the record was dropped from the review.

We searched Pubmed with the following query:

(((TB or Tuberculosis)) AND (ARI OR "Annual Risk of Infection" OR LTBI OR "latent tuberculosis infection")) AND (Survey OR population OR community OR representative) Filters: Publication date from 1990/01/01 to 2017/12/31

This yielded 1159 hits (query run on 10^th^ March 2016). In addition, 14 (Bourdin-Trunz *et al.*)+ 7 (van Leth) = 21 potential records were considered.

Paper selection and data extraction was done by RMGJH. Forty-three papers were included for full review, of which 23 contributed data. The selection process, and reasons for exclusion are shown in Figure 1. Papers were evaluated for bias with regard to representativeness of their sampling by reviewing the methods section, but not for methods used in the TST survey. This was in line with the other reviews that provided data. To reduce the risk of overestimation of infection prevalence due to BCG, we recorded the ARI estimate from BCG-scar negative individuals where possible.

Each study provided a single data point and confidence interval for a specific country to inform the ARI model, there was no attempt or need to combine results across studies, or do further analysis. This was in line with the other reviews that provided data

No funding was provided for this review.

Full-text articles excluded, with reasons
(n = 20)

Data not available n = 11

Overlap with other paper n = 7

Not nationally representative n = 3

Studies included in qualitative synthesis
(n = 23)

Full-text articles assessed for eligibility
(n = 43)

Records excluded
(n = 1128)

Records screened
(n = 1171)

Records after duplicates removed
(n = 1171)

Additional records identified through other sources
(n = 21)

Records identified through database searching
(n = 1159)

Figure A: PRISMA flow chart for systematic review

### References from review (ordered by time of publication)

1. Azbite M. Tuberculin survey in Ethiopia. Kekkaku : [Tuberculosis]. 1992;67(8):539-44. Epub 1992/08/01. PubMed PMID: 1405182.

2. Bouros D, Demoiliopoulos I, Moschos M, Panagou P, Demoiliopoulos D, Konstantopoulos S, et al. Tuberculin sensitivity trends in Hellenic army recruits during the period 1981-91. Tubercle and lung disease : the official journal of the International Union against Tuberculosis and Lung Disease. 1995;76(2):126-9. Epub 1995/04/01. PubMed PMID: 7780094.

3. Champetier de Ribes G, Ranaivoson G, Rakotoherisoa E, Andriamahefazafy B, Blanchy S. [Annual risk of tuberculosis infection in Madagascar: study from 1991 to 1994]. Bulletin de la Societe de pathologie exotique (1990). 1997;90(5):349-52. Epub 1997/01/01. PubMed PMID: 9507769.

4. Bosman MC, Swai OB, Kwamanga DO, Agwanda R, Idukitta G, Misljenovic O. National tuberculin survey of Kenya, 1986-1990. The international journal of tuberculosis and lung disease : the official journal of the International Union against Tuberculosis and Lung Disease. 1998;2(4):272-80. Epub 1998/04/29. PubMed PMID: 9559397.

5. Hong YP, Kim SJ, Lew WJ, Lee EK, Han YC. The seventh nationwide tuberculosis prevalence survey in Korea, 1995. The international journal of tuberculosis and lung disease : the official journal of the International Union against Tuberculosis and Lung Disease. 1998;2(1):27-36. PubMed PMID: 9562108.

6. El Ibiary S, de Coster EJ, Tolba FM, van Maaren P, Wasily L, van Cleeff M, et al. Trend in the annual risk of tuberculous infection in Egypt, 1950-1996. The international journal of tuberculosis and lung disease : the official journal of the International Union against Tuberculosis and Lung Disease. 1999;3(4):294-9. Epub 1999/04/17. PubMed PMID: 10206499.

7. Odhiambo JA, Borgdorff MW, Kiambih FM, Kibuga DK, Kwamanga DO, Ng'ang'a L, et al. Tuberculosis and the HIV epidemic: increasing annual risk of tuberculous infection in Kenya, 1986-1996. American journal of public health. 1999;89(7):1078-82. Epub 1999/07/08. PubMed PMID: 10394319; PubMed Central PMCID: PMCPmc1508825.

8. Tupasi TE, Radhakrishna S, Pascual ML, Quelapio MI, Villa ML, Co VM, et al. BCG coverage and the annual risk of tuberculosis infection over a 14-year period in the Philippines assessed from the Nationwide Prevalence Surveys. The international journal of tuberculosis and lung disease : the official journal of the International Union against Tuberculosis and Lung Disease. 2000;4(3):216-22. Epub 2000/04/06. PubMed PMID: 10751066.

9. Arnadottir TH, Soukaseum H, Vangvichit P, Bounmala S, Vos E. Prevalence and annual risk of tuberculosis infection in Laos. The international journal of tuberculosis and lung disease : the official journal of the International Union against Tuberculosis and Lung Disease. 2001;5(5):391-9. Epub 2001/05/05. PubMed PMID: 11336268.

10. Tanzania Tuberculin Survey C. Tuberculosis control in the era of the HIV epidemic: risk of tuberculosis infection in Tanzania, 1983-1998. The international journal of tuberculosis and lung disease : the official journal of the International Union against Tuberculosis and Lung Disease. 2001;5(2):103-12. PubMed PMID: 11258503.

11. Norval PY, Roustit C, San KK. From tuberculin to prevalence survey in Cambodia. The international journal of tuberculosis and lung disease : the official journal of the International Union against Tuberculosis and Lung Disease. 2004;8(3):299-305. Epub 2004/05/14. PubMed PMID: 15139467.

12. Salaniponi FM, Kwanjana J, Veen J, Misljenovic O, Borgdorff MW. Risk of infection with Mycobacterium tuberculosis in Malawi: national tuberculin survey 1994. The international journal of tuberculosis and lung disease : the official journal of the International Union against Tuberculosis and Lung Disease. 2004;8(6):718-23. Epub 2004/06/09. PubMed PMID: 15182141.

13. Egwaga SM, Cobelens FG, Muwinge H, Verhage C, Kalisvaart N, Borgdorff MW. The impact of the HIV epidemic on tuberculosis transmission in Tanzania. AIDS (London, England). 2006;20(6):915-21. Epub 2006/03/22. doi: 10.1097/01.aids.0000218557.44284.83. PubMed PMID: 16549977.

14. Begum V, van der Werf MJ, Becx-Bleumink M, Borgdorff MW. Viewpoint: do we have enough data to estimate the current burden of tuberculosis? The example of Bangladesh. Tropical medicine & international health : TM & IH. 2007;12(3):317-22. Epub 2007/02/23. doi: 10.1111/j.1365-3156.2006.01790.x. PubMed PMID: 17313503.

15. Shrestha KB, Malla P, Jha KK, Shakya TM, Akhtar M, Gunneberg C, et al. First national tuberculin survey in Nepal. The international journal of tuberculosis and lung disease : the official journal of the International Union against Tuberculosis and Lung Disease. 2008;12(8):909-15. Epub 2008/07/24. PubMed PMID: 18647450.

16. Al-Absi A, Bassili A, Abdul Bary H, Barker A, Daniels M, Munim A, et al. The decline of tuberculosis in Yemen: evaluation based on two nationwide tuberculin surveys. The international journal of tuberculosis and lung disease : the official journal of the International Union against Tuberculosis and Lung Disease. 2009;13(9):1100-5. Epub 2009/09/03. PubMed PMID: 19723398.

17. Kwamanga D, Chakaya J, Sitienei J, Kalisvaart N, L'Herminez R, van der Werf MJ. Tuberculosis transmission in Kenya: results of the third National Tuberculin Survey. The international journal of tuberculosis and lung disease : the official journal of the International Union against Tuberculosis and Lung Disease. 2010;14(6):695-700. Epub 2010/05/22. PubMed PMID: 20487606.

18. Chadha VK, Sarin R, Narang P, John KR, Chopra KK, Jitendra R, et al. Trends in the annual risk of tuberculous infection in India. The international journal of tuberculosis and lung disease : the official journal of the International Union against Tuberculosis and Lung Disease. 2013;17(3):312-9. Epub 2013/01/17. doi: 10.5588/ijtld.12.0330. PubMed PMID: 23321394.

19. Hoa NB, Cobelens FG, Sy DN, Nhung NV, Borgdorff MW, Tiemersma EW. First national tuberculin survey in Viet Nam: characteristics and association with tuberculosis prevalence. The international journal of tuberculosis and lung disease : the official journal of the International Union against Tuberculosis and Lung Disease. 2013;17(6):738-44. Epub 2013/05/17. doi: 10.5588/ijtld.12.0200. PubMed PMID: 23676155.

20. Hossain S, Zaman K, Banu S, Quaiyum MA, Husain MA, Islam MA, et al. Tuberculin survey in Bangladesh, 2007-2009: prevalence of tuberculous infection and implications for TB control. The international journal of tuberculosis and lung disease : the official journal of the International Union against Tuberculosis and Lung Disease. 2013;17(10):1267-72. Epub 2013/09/13. doi: 10.5588/ijtld.13.0114. PubMed PMID: 24025376.

21. Wangchuk LZ, Chadha VK. Annual risk of tuberculous infection among schoolchildren in Bhutan. The international journal of tuberculosis and lung disease : the official journal of the International Union against Tuberculosis and Lung Disease. 2013;17(4):468-72. Epub 2013/03/15. doi: 10.5588/ijtld.12.0668. PubMed PMID: 23485380.

22. Adetifa IM, Muhammad AK, Jeffries D, Donkor S, Borgdorff MW, Corrah T, et al. A Tuberculin Skin Test Survey and the Annual Risk of Mycobacterium tuberculosis Infection in Gambian School Children. PloS one. 2015;10(10):e0139354. Epub 2015/10/16. doi: 10.1371/journal.pone.0139354. PubMed PMID: 26465745; PubMed Central PMCID: PMCPmc4605652.

23. Minime-Lingoupou F, Ouambita-Mabo R, Komangoya-Nzozo AD, Senekian D, Bate L, Yango F, et al. Current tuberculin reactivity of schoolchildren in the Central African Republic. BMC public health. 2015;15:496. Epub 2015/05/20. doi: 10.1186/s12889-015-1829-8. PubMed PMID: 25981707; PubMed Central PMCID: PMCPmc4438344.

## Uncertainty in ARI estimates from TST surveys

ARI data were typically reported as a point estimate, together with the sample size and the mean age of participants. We show below how to use this information to construct an approximate data likelihood that conservatively represents the degree of precision of each study.

The force-of-infection, $\lambda$, for a given study is effectively estimated from a binomial likelihood:

$$\prod_{i} Bin(N_{i},k_{i}|(1-e^{-\lambda a_{i}}))$$

where $N_{i},k_{i}$ are the number, and number infected in age category *i* and $a_{i}$ is the age of this category. The derivatives of the loglikelihood, *LL*, are

$$\frac{\partial LL}{\partial\lambda}= \sum_{i} \left\{ \frac{k_{i}a_{i}e^{-\lambda a_{i}}}{1-e^{-\lambda a_{i}}}-(N_{i}-k_{i})a_{i} \right\}$$

$$\frac{\partial^{2}LL}{\partial\lambda^{2}}= \sum_{i} -\frac{k_{i}a_{i}^{2}e^{\lambda a_{i}}}{{(e^{\lambda a_{i}}-1)}^{2}}$$

If $1-e^{-\lambda a}\approx\lambda a$ and $k_{i}\ll N_{i}$ then, writing $N=\sum_{i} N_{i}$ and $K=\sum_{i} k_{i}$, setting the derivative of the loglikelihood to zero gives

$$K\lesssim\lambda N\bar{a}$$

where $\bar{a}$ is the average age of the persons in the study. Similarly, the second equation becomes:

$$-\frac{\partial^{2}LL}{\partial\lambda^{2}}\approx\frac{K}{\lambda^{2}}\leq\frac{N\bar{a}}{\lambda}$$

so that this last quantity (i.e., ${N\bar{a}}/\lambda$) gives a conservative estimate of the precision associated with a given study.

## Data merging and special cases

24 countries were dropped as they could not be matched across the TB and population data (ISO3 codes: AIA AND ANT ASM BMU COK CYM DMA GRL KNA MCO MHL MNP MSR NIU NRU PLW SMR SXM TCA TKL TUV VGB WLF).

Because of the stochasticity in small populations, we dropped 24 countries with a population size below 500,000 (ISO3 codes: WSM ABW ATG BHS BLZ BRB BRN CUW FSM GRD GUM ISL KIR LCA MDV MLT NCL PYF STP SYC TON VCT VIR VUT). See Figure 2.

4 further countries were dropped because they had fewer than 15 data points in total (ISO3 codes: BES CUW SXM TLS).

Figure B: Ranked country sizes (500,000 population size cut-off as red line)

168 countries were left, with a total population in 2014 of 7,232,115 thousand (>99.9% of the estimated global population). (ISO3 codes: BWA BDI CMR ETH GMB LSO TZA ARG BRA IND IDN THA DZA AFG BHR KWT LBY PAK SYR CHN MYS KOR PHL AGO ALB ARE ARM AUS AUT AZE BEL BEN BFA BGD BGR BIH BLR BOL BTN CAF CAN CHE CHL CIV COD COG COL COM CPV CRI CUB CYP CZE DEU DJI DNK DOM ECU EGY ERI ESP EST FIN FJI FRA GAB GBR GEO GHA GIN GNB GNQ GRC GTM GUY HKG HND HRV HTI HUN IRL IRN IRQ ISR ITA JAM JOR JPN KAZ KEN KGZ KHM LAO LBN LBR LKA LTU LUX LVA MAC MAR MDA MDG MEX MKD MLI MMR MNE MNG MOZ MRT MUS MWI NAM NER NGA NIC NLD NOR NPL NZL OMN PAN PER PNG POL PRI PRK PRT PRY PSE QAT ROU RUS RWA SAU SDN SEN SGP SLB SLE SLV SOM SRB SSD SUR SVK SVN SWE SWZ TCD TGO TJK TKM TTO TUN TUR UGA UKR URY USA UZB VEN VNM YEM ZAF ZMB ZWE).

For Sudan and South Sudan, we used prevalence estimates for Sudan prior to 2011 for both Sudan and South Sudan, and prevalence estimates specific to each country thereafter. For Serbia and Montenegro we used prevalence estimates reported for the combined country from 1990 to 2004 for the two countries separately, and prevalence estimates specific to each country thereafter.

## Modelling ARI from TB prevalence estimates

### The Styblo ratio

The Styblo ratio, $\beta$, between ARI and the prevalence of smear positive TB was taken to be distributed as

$$\beta\sim LogNormal(1.678,0.371)$$

following Dodd *et al.*

### The influence of HIV on smear-positivity

We followed the WHO assumptions for the durations of TB disease stratified by HIV- and notification-status. That is, we assumed these durations to be uniformly distributed

$$T_{1}^{n}\sim U[0.2,2]$$

$$T_{1}^{u}\sim U[1,4]$$

$$T_{2}^{n}\sim U[0.01,1]$$

$$T_{2}^{u}\sim U[0.01,0.22]$$

where 1 & 2 denote HIV negative & positive respectively, and *u* and *n* denote un-notified and notified cases, respectively. We used the WHO estimate of case detection ratio to compute the mean TB duration by HIV status for a given country-year

$$T_{1}=CDR.T_{1}^{n}+\left( 1-CDR \right).T_{1}^{u}$$

$$T_{2}=CDR.T_{2}^{n}+\left( 1-CDR \right).T_{2}^{u}$$

The smear-positivity of TB in people living with HIV was assumed to be a factor *f* less likely, where *f* is taken to be the ratio of two uniform distributions

$$f\sim U\left[ 0.3,0.4 \right]/U[0.4,0.5]$$

following Corbett *et al*.

The overall factor reduction *S* in smear-positivity for a country year then

$$S= \frac{(p.f.T_{2}+\left( 1-p \right).T_{1})}{(p.T_{2}+\left( 1-p \right).T_{1})}$$

where $p$ is the WHO estimate of the proportion of incident TB that is HIV-associated.

To assess the sensitivity or results to our assumption that the CDR is independent of HIV-status, we considered the change in the factor S if the CDR in people with HIV was 100%. The proportional change in S across country-years showed a median reduction of 0.14% (IQR: 0.04% - 0.55%).

### The influence of age mix on smear-positivity

The proportions $p_{1}$, $p_{2}$ and $p_{3}$ of TB in 0-4, 5-14 and $\geq$15 age groups was computed using the model of Dodd *et al.* The fractions $f_{1}$, $f_{2}$ and $f_{3}$ smear positive in these age groups were taken from the systematic review and meta-analysis of Kunkel *et al.*. The overall fraction smear positive was then

$$p_{1}.f_{1}+ p_{2}.f_{2} {+ p}_{3}.f_{3}$$

### Propagation of uncertainty

The uncertainty of all distributions, including those characterising the uncertainty in TB prevalence estimates, CDR, proportion of TB that in PLHIV, disease durations and proportions smear-positive were propagated through to give an estimate of ARI variance using the delta method. The validity of this approximation was checked by simulation for a data subset (not shown). The largest contributors to uncertainty in ARI from this procedure were uncertainties in the prevalence estimates and the Styblo ratio.

## Gaussian Process calculations

We took the log of ARI to follow a Gaussian process

$$\log\left( {ARI}_{t} \right)\mathcal{\sim GP(}m\left( t \right),k(t,t'))$$

where $m\left( t \right)=c_{0}+c_{1}.t$ for the linear trends analysis and simply a constant for the flat trends sensitivity analysis. We assumed vague priors for these coefficients, and used a squared exponential kernel to model the covariance

$$k\left( t,t^{'} \right)=\sigma_{k}^{2}.exp \left( -\frac{\left( t-t^{'} \right)^{2}}{2\mathcal{l}^{2}} \right)$$

with normal priors on the hyper-parameters $log(\sigma_{k})$ and $log\mathcal{(l)}$, with standard deviations of 100 and means corresponding to 0.5 for the overall kernel scale and 2 years for the smoothing time scale, respectively.

For the observed log ARI values, $y_{t}$, we assumed that

$$cov\left( y_{t},y_{t^{'}} \right)= k\left( t,t^{'} \right)+ \sigma_{t}^{2}.\delta_{t,t'}^{2}$$

where ${1/\sigma}_{t}^{2}$ specifies the measurement precision, as calculated by propagation of errors for data points derived from prevalence estimates, and using the method of the previous section of data points derived from TST surveys.

Hyper-parameters were set gradient-based optimisation of the posterior, i.e. the sum of the log marginal likelihood (Equation 2.45 of Rasmussen *et al.*) and the log priors specified above. Models were visually assessed for fit to data mid-points and uncertainty.

## Modelling LTBI burden given ARI

### All M.tb infection

Let $\lambda_{t}$ be a sampled ARI through calendar time for a particular country.

$$H_{a}=\int_{0}^{a} {da.\lambda}_{a}$$

The cumulative hazard, $H_{a}$, of infection for those of age $a$ is

$$H_{a}=\sum_{i=0}^{a} \lambda_{2015-i}$$

The proportion of this age group that is latently infected is therefore

$$P_{a}=1-exp(-H_{a})$$

Given that in this country the number of people who are age $a$ in 2014 is $N_{a}$, the number who are latently infected in each age group $L_{a}$ is just

$$L_{a}=P_{a}N_{a}=(1-\exp\left( -H_{a} \right))N_{a}$$

Now in fact we are only provided with demographic information in 5-year age bins, $M_{A}$ for a particular age group $A$. We therefore have

$$L_{A}=\sum_{a\in A} P_{a}N_{a}= M_{A}\times\frac{1}{5}\sum_{a\in A} P_{a}$$

### Infection with M.tb for the first time within 2 years

Let $T=2$years. The probability of infection for the first time within 2 years is given by

$$P_{a}^{<T}=\exp\left( -H_{a-T} \right)-\exp\left( -H_{a} \right)$$

where we define $H_{a}=0$ when $a<0$.

### Infection or re-infection with M.tb within 2 years

The hazard ratio protection $\alpha$ was characterized as

$$\alpha\sim Beta(77.88,20.70)$$

to match the mean and 95% confidence interval reported in Andrews *et al.* The incidence of infection for a given age is then

$$I_{a}=\lambda_{a}\left( \alpha(1-\exp\left( -H_{a} \right))+exp(-H_{a}) \right)$$

where $\lambda_{a}$ is the force-of-infection experienced by a cohort alive now when they were age $a$, which integrates to give

$$P_{a}^{<T}=\alpha{(H}_{a}-H_{a-T})+(1-\alpha)\left( e^{-H_{a-T}}-e^{-H_{a}} \right)$$

where $H_{a}$ is defined to be 0 when $a<0$.

Code for calculations can be found at <https://github.com/petedodd/LTBIest>.

# Results

## Gaussian process regressions for ARI with linear trends

Figure C: WHO AFR region

Figure D: WHO AMR region

Figure E: WHO EMR region

Figure F: WHO EUR region

Figure G: WHO SEA region

Figure H: WHO WPR region

## Top 20 countries by absolute LTBI burden (figure) and country level results (file)

Figure I: Top 20 countries by number of latent *M.tb* infections (color shows percentage prevalence). Country level results can be viewed in the supporting file individual_country_ests.csv

## Recent infection and re-infection by age

Figure J: Recent infection and re-infection by age under base case assumption around protection from re-infection.

## Gaussian process regressions for ARI with flat trends

Figure K: WHO AFR region (flat trends)

Figure L: WHO AMR region (flat trends)

Figure M: WHO EMR region (flat trends)

Figure N: WHO EUR region (flat trends)

Figure O: WHO SEA region (flat trends)

Figure P: WHO WPR region (flat trends)

## Supporting tables

Table A: Proportions with LTBI for flat trend sensitivity analysis

| **WHO region** | **All LTBI** | | **Recent infection prevalence (within 2 years)** | |
| --- | --- | --- | --- | --- |
|  | **Prevalence (%)** | **Proportion of infections in children <15 years (%)** | **(%)** | **Proportion with INH-R infection (%)** |
| AFR | 20.8 [19.4 - 22.6] | 14.4 [13.4 - 15.3] | 1.5 [1.3 - 1.8] | 7.4 [6.5 - 8.7] |
| AMR | 5.2 [4.5 - 6.2] | 5.0 [4.1 - 5.7] | 0.2 [0.2 - 0.3] | 7.1 [6.1 - 9.0] |
| SEA | 30.3 [27.1 - 35.2] | 7.6 [6.6 - 8.5] | 1.2 [1.0 - 1.7] | 9.6 [8.8 - 10.3] |
| EMR | 13.3 [11.7 - 16.0] | 9.8 [8.3 - 11.2] | 0.7 [0.6 - 1.0] | 13.3 [9.9 - 15.6] |
| WPR | 18.7 [14.8 - 26.8] | 3.7 [2.7 - 4.5] | 0.6 [0.4 - 0.8] | 14.8 [13.9 - 15.7] |
| EUR | 8.2 [7.0 - 10.1] | 3.3 [2.7 - 3.8] | 0.3 [0.2 - 0.3] | 28.7 [23.0 - 44.1] |
| **GLOBAL** | **18.5 [17.0 - 20.7]** | **7.4 [6.5 - 7.9]** | **0.8 [0.7 - 1.0]** | **11.0 [10.3 - 12.0]** |

Table B: Proportions with LTBI for 50% protection sensitivity analysis

| **WHO region** | **All LTBI** | | **Recent infection prevalence (within 2 years)** | |
| --- | --- | --- | --- | --- |
|  | **Prevalence (%)** | **Proportion of infections in children <15 years (%)** | **(%)** | **Proportion with INH-R infection (%)** |
| AFR | 22.4 [20.6 - 24.6] | 13.3 [11.8 - 14.6] | 1.6 [1.4 - 1.9] | 7.4 [6.5 - 8.8] |
| AMR | 11.0 [7.0 - 20.0] | 2.3 [1.3 - 3.7] | 0.2 [0.2 - 0.2] | 7.1 [6.2 - 8.7] |
| SEA | 30.8 [28.3 - 34.8] | 7.4 [6.3 - 8.2] | 1.4 [1.0 - 1.8] | 9.5 [8.7 - 10.4] |
| EMR | 16.3 [13.4 - 20.5] | 7.9 [6.0 - 9.4] | 0.8 [0.5 - 1.1] | 13.2 [10.1 - 15.5] |
| WPR | 27.9 [19.3 - 40.1] | 2.4 [1.7 - 3.5] | 0.6 [0.4 - 0.8] | 14.7 [13.8 - 15.6] |
| EUR | 13.7 [9.8 - 19.8] | 2.0 [1.3 - 2.7] | 0.3 [0.2 - 0.4] | 29.1 [23.9 - 48.1] |
| **GLOBAL** | **23.0 [20.4 - 26.4]** | **5.9 [5.1 - 6.7]** | **0.9 [0.7 - 1.0]** | **11.0 [10.2 - 12.0]** |

Table C: Numbers (thousands) of infections for flat trend sensitivity analysis

| **WHO region** | **All LTBI** | | **Recent infection prevalence (within 2 years)** | |
| --- | --- | --- | --- | --- |
|  | **Number (K)** | **Number (K) of infections in children <15 years** | **Number (K)** | **Number (K) with INH-R infection** |
| AFR | 200,000 [187,000 - 218,000] | 28,700 [26,700 - 31,600] | 14,400 [12,500 - 17,000] | 1,070 [863 - 1,350] |
| AMR | 51,100 [44,400 - 60,800] | 2,550 [ 2,310 - 2,800] | 1,990 [ 1,580 - 2,520] | 143 [105 - 185] |
| SEA | 577,000 [516,000 - 670,000] | 43,700 [38,800 - 49,800] | 23,400 [18,900 - 32,600] | 2,230 [1,730 - 3,180] |
| EMR | 84,800 [74,300 - 102,000] | 8,240 [ 7,220 - 9,520] | 4,760 [ 3,710 - 6,300] | 631 [418 - 899] |
| WPR | 344,000 [272,000 - 493,000] | 12,700 [11,300 - 14,500] | 10,300 [ 7,960 - 14,200] | 1,540 [1,130 - 2,140] |
| EUR | 74,600 [63,300 - 91,500] | 2,450 [ 2,220 - 2,710] | 2,360 [ 1,970 - 3,060] | 702 [516 - 1,110] |
| **GLOBAL** | **1,340,000 [1,230,000 - 1,500,000]** | **98,600 [93,100 - 106,000]** | **57,700 [51,700 - 68,800]** | **6,390 [5,620 - 7,660]** |

Table D: Numbers (thousands) of infections for 50% protection sensitivity analysis

| **WHO region** | **All LTBI** | | **Recent infection prevalence (within 2 years)** | |
| --- | --- | --- | --- | --- |
|  | **Number (K)** | **Number of infections in children <15 years** | **Number (K)** | **Number (K) with INH-R infection** |
| AFR | 216,000 [198,000 - 237,000] | 28,700 [26,700 - 30,800] | 15,600 [13,300 - 18,200] | 1,160 [ 943 - 1,410] |
| AMR | 108,000 [68,900 - 196,000] | 2,470 [ 2,240 - 2,710] | 1,860 [ 1,530 - 2,360] | 134 [ 101 - 179] |
| SEA | 587,000 [540,000 - 662,000] | 43,300 [38,700 - 48,300] | 26,100 [19,300 - 35,200] | 2,510 [ 1,850 - 3,370] |
| EMR | 104,000 [85,200 - 130,000] | 8,060 [ 7,090 - 9,240] | 4,940 [ 3,460 - 6,860] | 633 [ 433 - 891] |
| WPR | 514,000 [356,000 - 739,000] | 12,400 [10,900 - 13,800] | 10,300 [ 7,750 - 14,100] | 1,520 [ 1,130 - 2,140] |
| EUR | 124,000 [89,100 - 180,000] | 2,430 [ 2,220 - 2,690] | 2,420 [ 1,980 - 3,300] | 712 [ 516 - 1,260] |
| **GLOBAL** | **1,660,000 [1,480,000 - 1,910,000]** | **97,100 [91,700 - 103,000]** | **61,900 [53,400 - 71,300]** | **6,740 [ 5,820 - 8,030]** |

## Recent infection and re-infection by age (sensitivity analysis)

Figure Q: Sensitivity analysis 50% protection against re-infection

## Conceptual overview

Figure R: Conceptual overview
